# Stress tolerance in diapausing embryos of *Artemia franciscana* is dependent on heat shock factor 1 (Hsf1)

**DOI:** 10.1371/journal.pone.0200153

**Published:** 2018-07-06

**Authors:** Jiabo Tan, Thomas H. MacRae

**Affiliations:** Department of Biology, Dalhousie University, Halifax, N. S., Canada; Universite de Liege, BELGIUM

## Abstract

Embryos of the crustacean, *Artemia franciscana*, may undergo oviparous development, forming encysted embryos (cysts) that are released from females and enter diapause, a state of suppressed metabolism and greatly enhanced stress tolerance. Diapause-destined embryos of *A*. *franciscana* synthesize three small heat shock proteins (sHsps), p26, ArHsp21 and ArHsp22, as well as artemin, a ferritin homologue, all lacking in embryos that develop directly into nauplii. Of these diapause-specific molecular chaperones, p26 and artemin are important contributors to the extraordinary stress tolerance of *A*. *franciscana* cysts, but how their synthesis is regulated is unknown. To address this issue, a cDNA for heat shock factor 1 (Hsf1), shown to encode a protein similar to Hsf1 from other organisms, was cloned from *A*. *franciscana*. Hsf1 was knocked down by RNA interference (RNAi) in nauplii and cysts of *A*. *franciscana*. Nauplii lacking Hsf1 died prematurely upon release from females, showing that this transcription factor is essential to the survival of nauplii. Diapause cysts with diminished amounts of Hsf1 were significantly less stress tolerant than cysts containing normal levels of Hsf1. Moreover, cysts deficient in Hsf1 possessed reduced amounts of p26, ArHsp21, ArHsp22 and artemin, revealing dependence on Hsf1 for expression of their genes and maximum stress tolerance. The results demonstrate an important role for Hsf1, likely in concert with other transcription factors, in the survival and growth of *A*. *franciscana* and in the developmentally regulated synthesis of proteins responsible for the stress tolerance of diapausing *A*. *franciscana* cysts.

## Introduction

Ovoviviparously developing embryos of the extremophile crustacean, *A*. *franciscana*, are released from females as swimming nauplii whereas oviparously developing embryos stall at gastrulation and enclose within a chitinous shell, forming cysts [1–3]. Upon release from females, cysts enter diapause, a phylogenetically wide spread state of metabolic depression and enhanced stress tolerance [3–10]. *A*. *franciscana* cysts remain in diapause, even in favorable environments, until encountering diapause terminating signals such as desiccation and/or cold [11]. Upon diapause termination, cysts resume development, but if conditions are not conducive to growth, they undergo quiescence, remaining dormant and stress tolerant until adequate moisture, oxygen and temperature prevail.

*Artemia* cysts possess several adaptations that promote survival during diapause and quiescence when they are exposed to stressors such as temperature extremes, desiccation, anoxia and radiation. Adaptations include a rigid, semi-permeable shell [3, 12, 13], high trehalose concentration [14–16] late embryogenesis abundant (LEA) proteins [17–22], and the abundant, diapause-specific, ATP-independent molecular chaperones, p26, a sHsp [1, 23–26] and artemin, a ferritin homologue [24, 27–29]. *A*. *franciscana* cysts contain at least two other sHsps, ArHsp21 and ArHsp22, which are synthesized only in embryos that enter diapause but they have a limited role in stress tolerance [26, 30, 31]. Of the four known developmentally regulated ATP-independent molecular chaperones synthesized in diapause-destined *A*. *franciscana* embryos only ArHsp22 is induced by stress, and then only in adults [31]. Control of the synthesis of these proteins has not otherwise been explored.

Contrasting the situation in *A*. *franciscana*, the regulation of sHsp synthesis has been studied extensively in *Drosophila melanogaster*, an insect with a weak diapause. Of the twelve sHsp genes in *D*. *melanogaster*, those encoding Hsp22, Hsp23, Hsp26 and Hsp27 are stress inducible and developmentally regulated, with the synthesis of each sHsp specific to life history stage and cell type [32–37]. Expression of the genes encoding these sHsps in *D*. *melanogaster* depends upon a constitutively synthesized but stress inducible transcription factor termed heat shock factor (Hsf). Only one Hsf is found in *D*. *melanogaster* and lower eukaryotes, whereas other eukaryotic organisms contain multiple Hsfs [38–42].

The domain structure of Hsf1 is well conserved across species. The winged helix-turn-helix DNA-binding domain (DBD) at the amino-terminus is separated by a linker region from a coiled-coil hydrophobic oligomerization region containing two heptad repeat domains named HR-A and HR-B. The oligomerization domain is followed by a variable domain that negatively regulates the transactivation domain (TAD) and a short heptad repeat domain called HR-C that interacts with the oligomerization domain to prevent spontaneous trimer formation, although not all Hsf1s contain an HR-C domain. TAD, localized at the carboxyl terminus and divided into subdomains TAD1 and TAD2, is modified post-translationally which affects gene recognition and Hsf1 activation. The DNA-binding and HR-A/B domains of Hsf1 are similar in sequence from one organism to another [38–43].

Hsf1 is activated by dissociation from molecular chaperones such as Hsp90/Hsp70, trimerization through intermolecular coiled-coil interactions of the HR domains, nuclear localization, and posttranslational modifications including SUMOylation, acetylation and phosphorylation [38, 39, 41, 43, 44]. Hsf1 binds to conserved upstream regulatory regions of genes termed heat shock elements (HSEs) with the coiled-coil domain wrapping around the DNA [45, 46]. HSEs are composed, with variation, of continuous, inverted, repeating units of the 5-base pair (bp) nucleotide sequence, 5’-nGAAn-3‘ where n is any nucleotide. There are often three repeating five bp units arranged tail to tail in a HSE with each unit capable of binding one Hsf1 in partnership with other proteins to regulate gene expression during stress [39, 41, 43, 47–50].

The objective of the study described herein was to determine if Hsf1 is required for stress tolerance in diapausing *A*. *franciscana* cysts and, if so, does this entail the regulation of genes encoding diapause-specific proteins. Consequently, *hsf1* cDNA was cloned from *A*. *franciscana* by rapid amplification of cDNA ends (RACE) and an antibody was made to a peptide deduced from the cDNA sequence. The cloned cDNA served as template for the production of double stranded RNA (dsRNA) employed to knockdown Hsf1 in *A*. *franciscana* cysts by RNAi. The Hsf1 knockdown cysts were less stress tolerant than cysts containing Hsf1 and they possessed reduced amounts of p26, ArHsp21, ArHsp22 and artemin. The results demonstrate that Hsf1 participates in the developmentally regulated expression of genes encoding molecular chaperones required for the survival of *A*. *franciscana* cysts during diapause.

## Results

### Nucleotide and amino acid sequences of *A*. *franciscana* Hsf1

**Fig 1:**
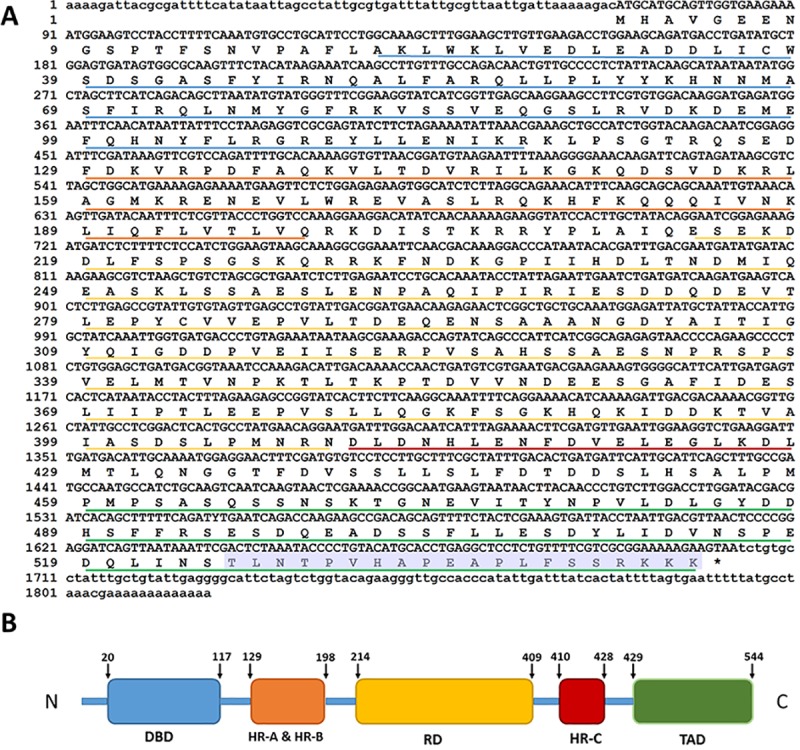
Hsf-1 sequence and domain structure. **(**A) *hsf1* cDNA and deduced amino acid sequences. DNA binding domain, blue underline; oligomerization domain; orange underline; regulatory domain, yellow underline; heptad repeat (HR-C) domain, red underline; transactivation domain, green underline. B. Schematic representation of Hsf1 domains in order from the amino to carboxyl terminal are DNA binding, DBD; oligomerization, HR-A & HR-B; regulatory (RD); heptad repeat, HR-C; trans-activation (TAD). The peptide 525-TLNTPVHAPEAPLFSSRKKK-544 was used as antigen to make an antibody to *A*. *franciscana* Hsf1 (shaded grey). *, stop codon. Nucleotide and amino acid residue positions are indicated on the left side of the figure. (B) Schematic representation of the domain structure of Hsf1 from *A*. *franciscana*.

**Fig 2:**
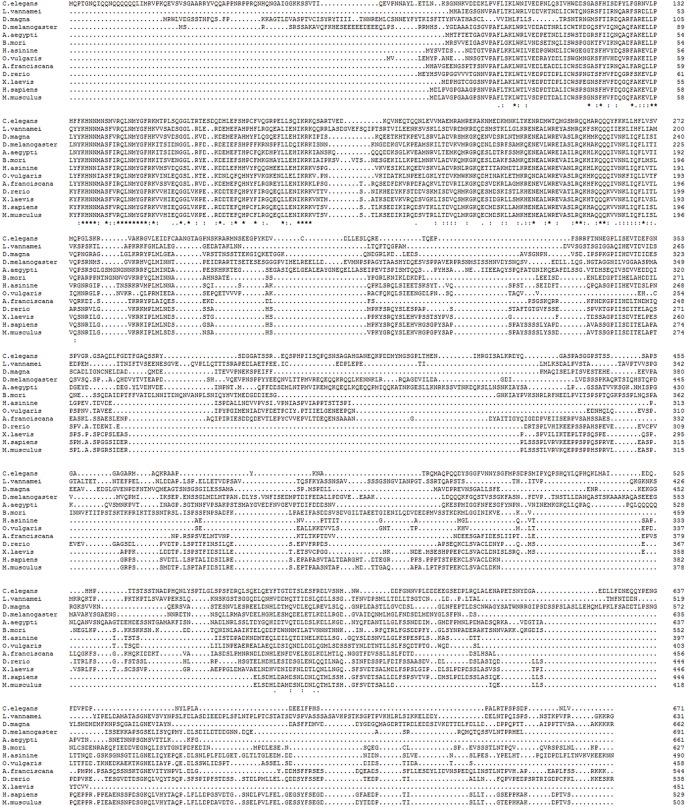
Multiple sequence alignment of Hsfs. The amino acid sequence of Hsf1 from *A*. *franciscana* was aligned with Hsf1s from the species listed in Table 1. Identical amino acid residues are indicated by an asterisk and similar amino acid residues by a colon. Amino acid residue positions are indicated on the right side of the figure.

A full-length *hsf1* cDNA (Accession Number KY985304) of 1818 nucleotides was obtained from nauplii of *A*. *franciscana* by RACE (Fig 1). The *A*. *franciscana hsf1* cDNA contained a 5’-untranslated region (UTR) of 78 nucleotides and a 3’-UTR of 115 nucleotides with a short poly(A) tail. The open reading frame (ORF) of 1632 bps encoded a polypeptide of 544 amino acid residues with a predicted molecular mass of 65.3 kDa and a theoretical pI of 4.74. Hsf1 from *A*. *franciscana* contained a typical amino-terminal DBD separated by a linker region from an oligomerization domain composed of two heptad repeat regions, HR-A & HR-B (Fig 1). Following the oligomerization domain was a regulatory domain (RD) that in Hsf1 from other species negatively influences the TAD, and a short heptad repeat, HR-C that regulates trimerization. The TAD resides at the carboxyl-terminus of *A*. *franciscana* Hsf1. A second *A*. *franciscana hsf1* cDNA with an identical ORF was cloned from a preparation of cyst RNA, thereby verifying the absence of PCR and sequencing artifacts (not shown). The DBD and HR-domains of these clones were similar in sequence to the corresponding domains from other organisms but sequence similarity in other regions was less evident (Fig 2; Table 1).

**Table 1:** Hsf1 from *A*. *franciscana* shares sequence identity with Hsf1 from other organisms.

Organism	Phylum	Class	Species	%Identity	Accession #
*Caenhorhabditis elegans*	Nematoda	Chromadorea	Roundworm	22.97	NP_493031.1
*Litopenaeus vannamei*	Arthropoda	Molacostraca	Shrimp	32.35	AHI13794.1
*Daphnia magna*	Arthropoda	Branchiopoda	Wafer flea	35.11	JAM73014.1
*Drosophila melanogaster*	Arthropoda	Insecta	Fruit fly	31.25	NP_476575.1
*Adedes aegypti*	Arthropoda	Insecta	Mosquito	30.33	JAN95553.1
*Bombyx mori*	Arthropoda	Insecta	Silkworm	30.88	BAK26396.1
*Haliotis asinina*	Mollusca	Gastropoda	Abalone	34.28	ABR15461.1
*Octopus vulgaris*	Mollusca	Cephalopoda	Octopus	34.06	AGZ63438.1
*Danio rerio*	Chordata	Actinopterygii	Zebrafish	32.89	NP_571675.1
*Xenopus laevis*	Chordata	Amphibia	Toad	37.91	NP_001084036.1
*Homo sapiens*	Chordata	Mammalia	Human	31.94	NP_005517.1
*Mus musculus*	Chordata	Mammalia	Mouse	33.2	NP_032322.1

The % identity between Hsf1 from *A*. *franciscana* and Hsf1s from other organisms was determined by SIAS at http://imed.med.ucm.es/Tools/sias.html.

### Knockdown of Hsf1 in *A*. *franciscana* cysts

**Fig 3:**
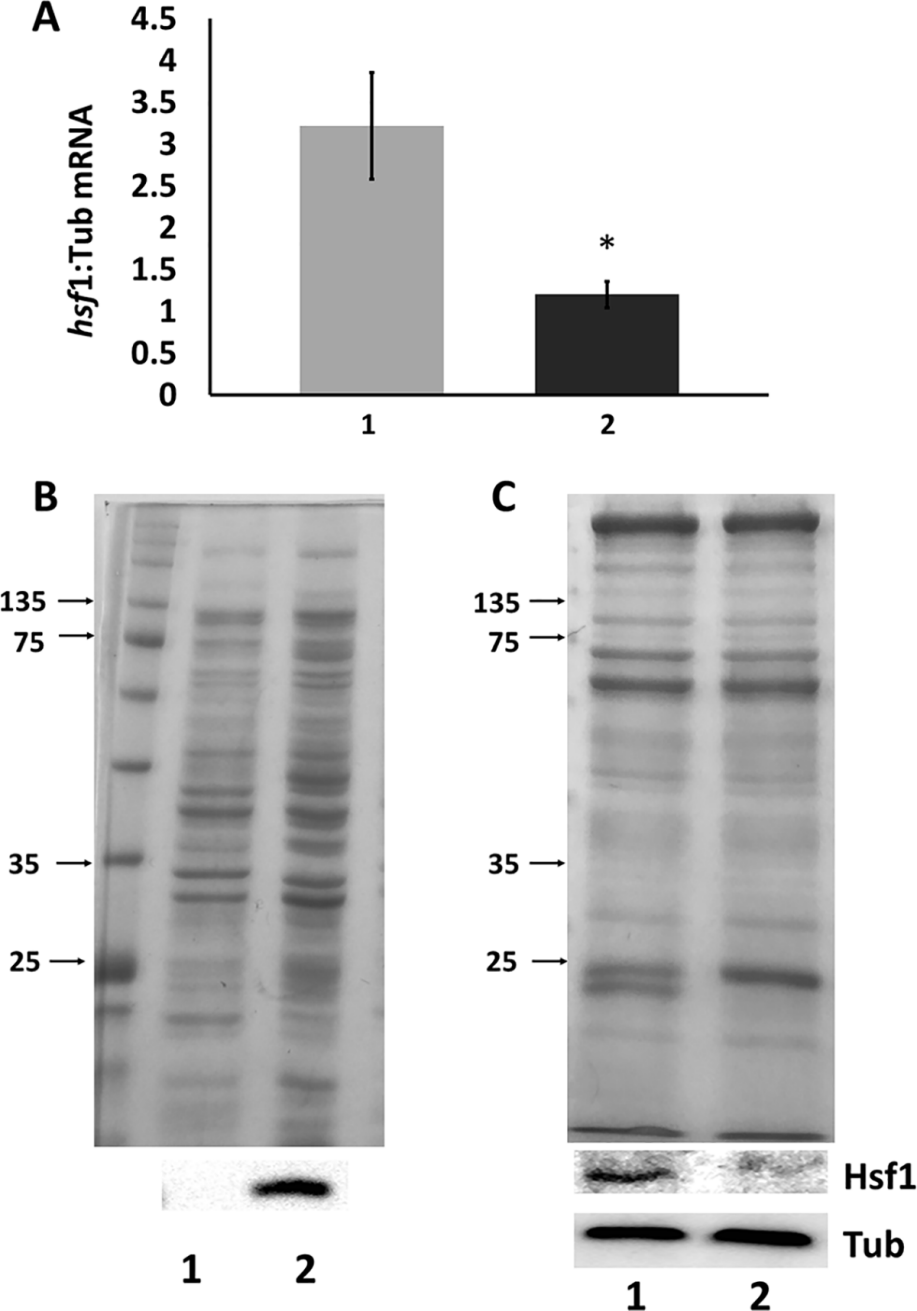
RNAi knocked down *hsf1* mRNA and protein in *A*. *franciscana* cysts. (A) The amount of *hsf1* mRNA in *A*. *franciscana* cysts released from females receiving *GFP* (1) and *hsf1* (2) dsRNA was determined by qPCR using tyrosinated α-tubulin mRNA as internal standard. The experiment was done 3 times and error bars show standard deviation. The asterisk indicates that the amount of *hsf1* mRNA in cysts from females receiving *hsf1* dsRNA was statistically different from the amount of *hsf1* mRNA in cysts from females receiving *GFP* dsRNA. (B) Protein extracts *from E*. *coli* transformed with expression plasmids either lacking (lane 1) or containing (lane 2) *hsf1* cDNA were resolved in SDS polyacrylamide gels and stained with Coomassie blue (upper) or blotted to nitrocellulose and reacted with antibody to Hsf1 (lower). (C) Protein extracts from 50 cysts released from females injected with either *GFP* dsRNA (1) or *hsf1* dsRNA (2) were resolved in SDS polyacrylamide gels and either stained with Coomassie blue (upper) or transferred to nitrocellulose and stained with antibody to Hsf1 or tubulin (Tub). The experiment was repeated several times and a representative example is shown.

As revealed by qRT-PCR with tyrosinated α-tubulin as internal standard the injection of *A*. *franciscana* females with *hsf1* dsRNA reduced *hsf1* mRNA in cysts by 64.3% in comparison to the amount of *hsf1* mRNA in cysts released from females injected with *GFP* dsRNA (Fig 3A).

The activity of an antibody raised to peptide 525-TLNTPVHAPEAPLFSSRKKK-544 from Hsf1 was confirmed by the probing of western blots containing protein extracts from ITPG-induced *E*. *coli* transformed with an expression plasmid containing an *hsf1* cDNA fragment encoding the peptide (Fig 3B). Immunoprobing of western blots containing protein extracts demonstrated that the amount of Hsf1 in cysts released from *A*. *franciscana* females injected with *hsf1* dsRNA was significantly reduced when compared to cysts from females receiving *GFP* dsRNA (Fig 3C). That equal amounts of protein, as determined by the Bradford assay, were applied to each well of SDS polyacrylamide gels was verified by Coomassie staining of gels and the probing of western blots for tyrosinated α-tubulin (Fig 3C).

### Knockdown of Hsf1 decreased the stress tolerance of *A*. *franciscana* cysts

**Fig 4:**
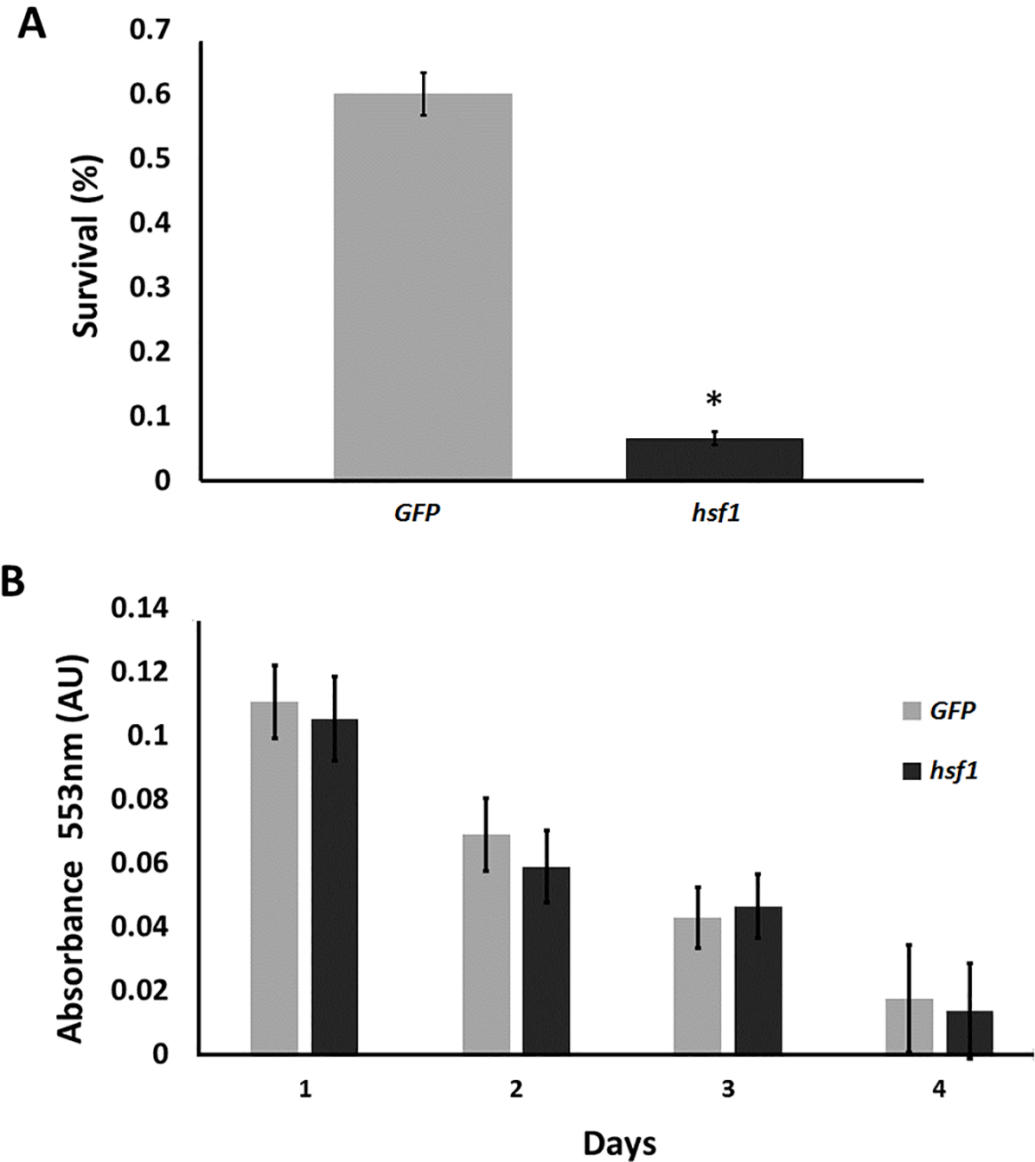
Knock down of Hsf1 reduced the stress tolerance of *A*. *franciscana* cysts. (A) Cysts released from females injected with dsRNA for GFP and Hsf1 were incubated in sea water for 7 days, desiccated, frozen and then incubated in sea water at room temperature to evaluate hatching. The experiment was done 3 times and error bars show standard deviation. The asterisk indicates that the survival of cysts from females receiving dsRNA for HSF1 was statistically different from the survival of cysts from females receiving *GFP* dsRNA. (B) To determine viability 10 cysts obtained from females injected with either *hsf1* or *GFP* dsRNA as indicated in the figure were incubated in test solution and the change in absorbance at 553 nm was measured immediately and on 3 consecutive days. The experiment was repeated 5 times and the standard error is shown.

*A*. *franciscana* cysts released from females injected with dsRNA for either GFP or Hsf1 and incubated 7 days in sea water were desiccated and frozen to simultaneously stress the organisms and terminate diapause, after which they were incubated in sea water at room temperature for hatching, the indicator of post-stress viability used herein. Approximately 60% of cysts from females containing normal amounts of Hsf1 hatched as compared to about 6% of cysts with reduced Hsf1 (Fig 4A).

Because visual inspection cannot reveal if knock down cysts are viable upon release from females they were examined for metabolic activity. The metabolic activities of cysts newly released from females injected with dsRNA for Hsf1 and GFP were very similar to one another and the metabolism of both groups of cysts declined to undetectable levels by 3–4 days as the cysts entered diapause (Fig 4B). Test solutions devoid of animals did not change in absorbance.

### Hsf1 knockdown reduced diapause-specific molecular chaperones in *A*. *franciscana* cysts

**Fig 5:**
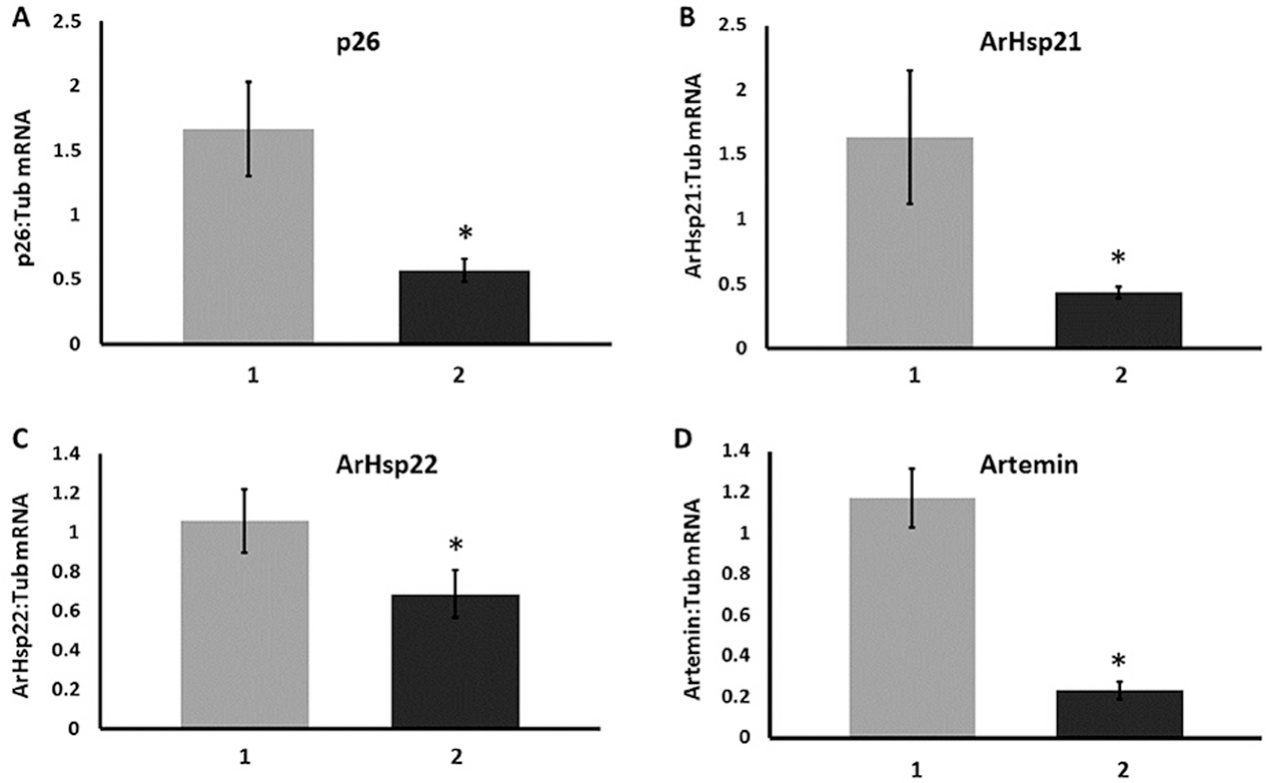
Knock down of Hsf1 reduced mRNA encoding diapause-specific molecular chaperones in *A*. *franciscana* cysts. RNA was prepared from cysts released from *A*. *franciscana* females injected with dsRNA for either GFP (1) or Hsf1 (2). mRNA for p26, artemin, ArHsp21 and ArHsp22, as indicated in the figure, was quantified by RT-qPCR using tyrosinated α-tubulin as internal standard. Experiments for each molecular chaperone were done 3 times and error bars show standard deviation. The asterisks indicate that the amount of molecular chaperone mRNA in cysts from females receiving dsRNA for Hsf1 was statistically different from the amount of molecular chaperone mRNA in cysts from females receiving *GFP* dsRNA.

Quantification of mRNA by qRT-PCR and normalization to α-tubulin mRNA revealed that cysts with reduced Hsf1 exhibited amounts of mRNA encoding the molecular chaperones p26, artemin, ArHsp21 and ArHsp22 that were respectively diminished by 65.3%, 80.4%, 73.7% and 35.0% (Fig 5).

**Fig 6:**
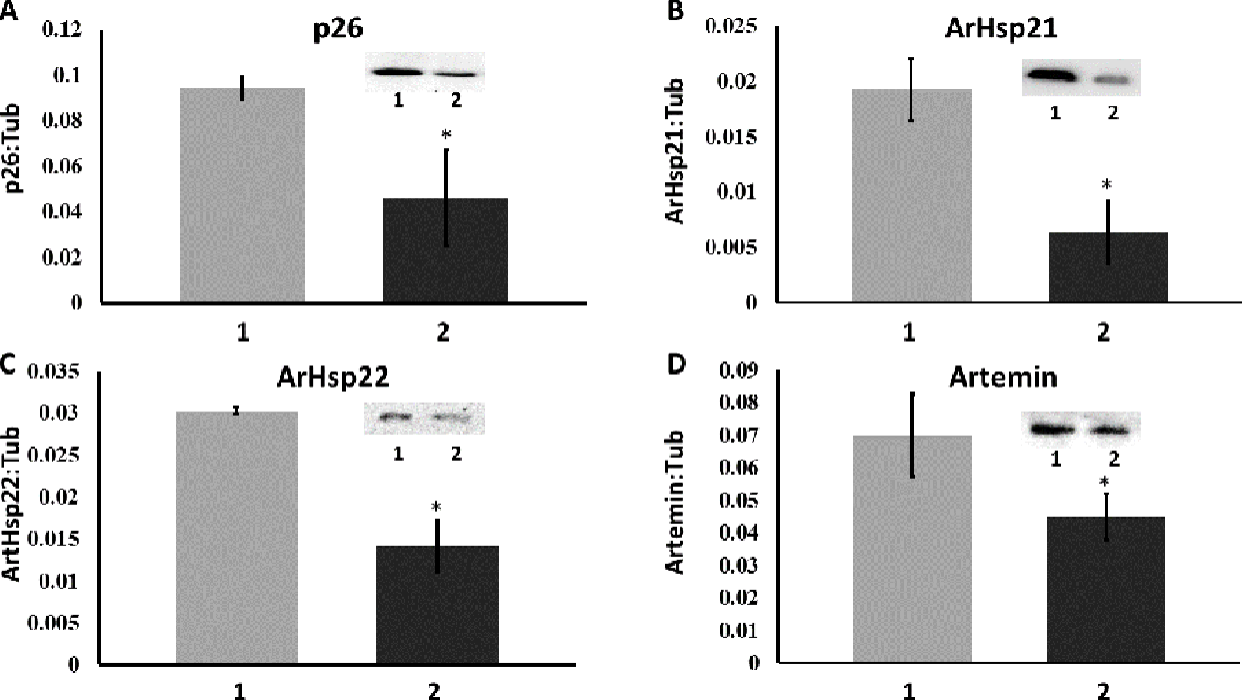
The knock down of Hsf1 reduced the amount of diapause-specific molecular chaperones *in A*. *franciscana* cysts. Protein extracts from 50 cysts released from females injected with either *GFP* dsRNA (1) or *hsf1* dsRNA (2) were resolved in SDS polyacrylamide gels, blotted to nitrocellulose and reacted with antibodies to p26, artemin, ArHsp21 and ArHsp22 as indicated in the figure. Insets, representative examples of western blots probed with antibodies to p26, artemin, ArHsp21, ArHsp22. The bands on the blots were quantified with Image Studio Software and the ratio of each molecular chaperone to tyrosinated α-tubulin was determined. Experiments for each molecular chaperone were done 2 times and error bars show standard deviation. The asterisks indicate that the amount of each molecular chaperone in cysts from females receiving dsRNA for Hsf1 was statistically different from the amount of that molecular chaperone in cysts from females receiving *GFP* dsRNA.

Immunoprobing of western blots demonstrated that *A*. *franciscana* cysts with reduced Hsf1 possessed diminished amounts of p26, artemin, ArHsp21, and ArHsp22 (Fig 6). Diapause-specific molecular chaperones always declined in cysts with reduced Hsf1 but the loss was not complete and the extent of knockdown varied from one experiment to another, ranging from 43% to 54% for p26, 52% to 74% for artemin, 31% to 34% for ArHsp21 and 44% to 50% for ArHsp22. That each gel lane of SDS polyacrylamide gels received similar amounts of protein, as determined by the Bradford assay, was confirmed by demonstrating the presence of equivalent amounts of tyrosinated α-tubulin in each lane (not shown).

### *A*. *franciscana* nauplii with depleted Hsf1 died prematurely

**Fig 7:**
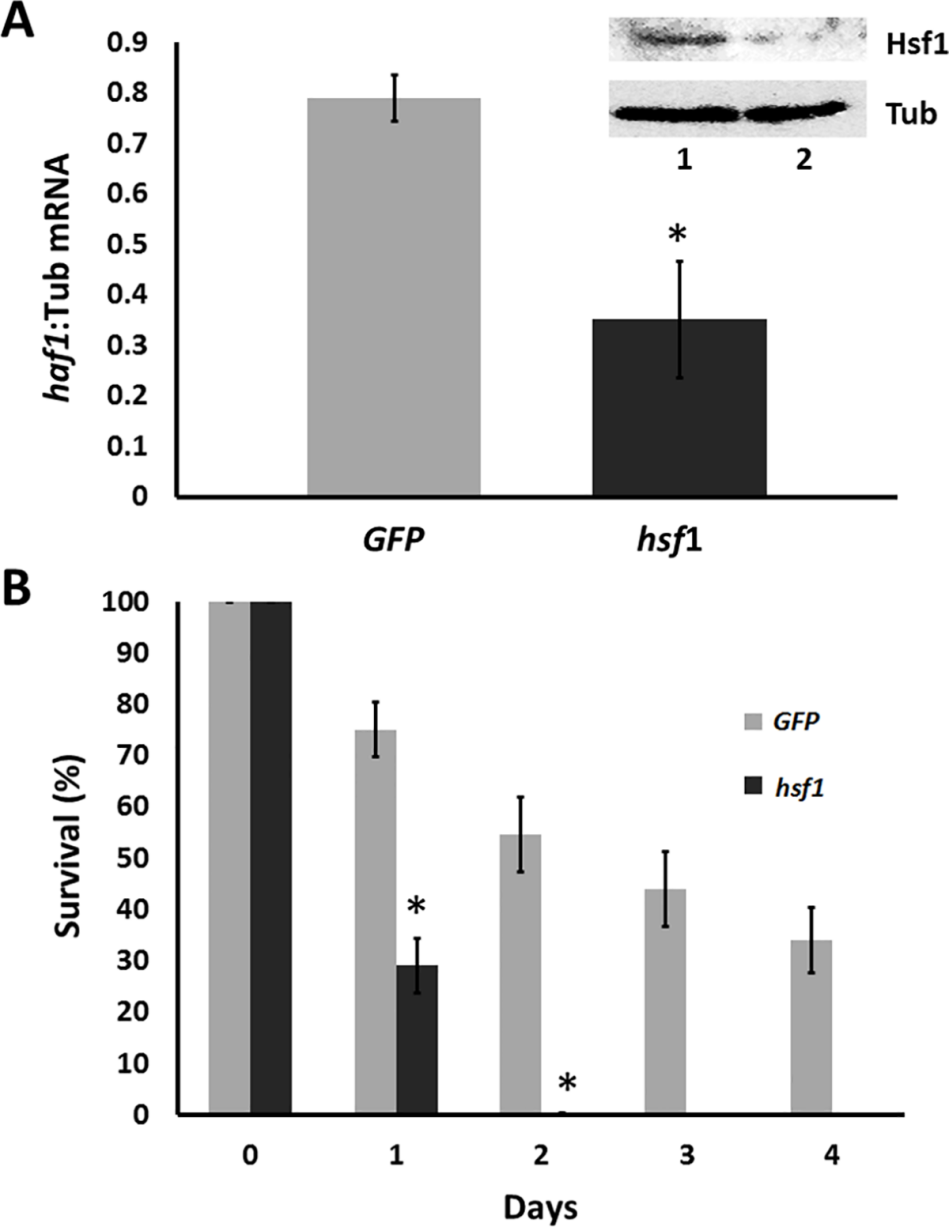
*A*. *franciscana* nauplii with depleted Hsf1 die prematurely. (A) The amount of *hsf1* mRNA in *A*. *franciscana* nauplii released by females receiving *hsf1* and *GFP* dsRNA was determined by qPCR using tyrosinated α-tubulin mRNA as internal standard. The asterisk indicates that the amount of *hsf1* mRNA in nauplii from females receiving dsRNA for Hsf1 was statistically different from the amount of *hsf1* mRNA in nauplii from females receiving *GFP* dsRNA. The results are based on three independent experiments and expressed as mean values ±S.D. Inset, protein extracts from 60 nauplii released from females injected with *GFP* dsRNA (1) and *hsf1* dsRNA (2) were resolved in SDS polyacrylamide gels, blotted to nitrocellulose and probed with antibodies to Hsf1 and tyrosinated α-tubulin as indicated in the figure. (B) Surviving (motile) nauplii released from females injected with either *GFP* or *Hsf1* dsRNA were counted daily and compared to the number of viable nauplii upon release. The experiment was done 3 times and error bars show standard deviation. The asterisks on days 1 and 2 indicate that the survival of nauplii released from females receiving *hsf1* dsRNA was statistically different from the survival of nauplii released from females receiving *GFP* dsRNA.

Nauplii obtained from females injected with dsRNA for Hsf1 exhibited reduced amounts of *hsf1* mRNA and protein in comparison to nauplii from females injected with dsRNA for GFP (Fig 7). Nonetheless, almost all nauplii either containing or lacking Hsf1 were, as indicated by swimming behavior, viable immediately after release (Fig 7). Approximately 70% of nauplii lacking Hsf1 died one day after release from females and all were dead within 2 days. In contrast, the death of nauplii containing Hsf1 occurred more slowly, with 75% of nauplii alive 1 day after release from females and 35% viable after 4 days when the experiment was terminated.

## Discussion

Hsf1 is activated during stress, modulating the expression of a variable repertoire of genes including those encoding molecular chaperones [51–53]. Biochemical and physiological changes associated with the stress response are an integral aspect of diapause and they entail modification of gene expression in response to environmental cues received prior to stress exposure. For example, the synthesis of Hsps is up-regulated in diapause-destined embryos of *A*. *franciscana* [1, 30, 31] and in other organisms undergoing diapause such as rotifers [54–55], copepods [56–57] and insects [8, 58–63], where they assist in maintaining protein homeostasis and stress tolerance. In spite of its importance in the regulation of molecular chaperone synthesis and stress tolerance Hsf1 function during diapause, a physiological adaptation to stress, has received limited attention.

That Hsf1 regulates Hsp synthesis during the development of diapause-destined *A*. *franciscana* embryos is indicated by HSEs in the upstream region of the diapause-specific gene encoding p26 [64]. The sHsp genes *AccHsp27*.*6*, *AccHsp24*.*2* and *AccHsp23*.*0* of the Chinese honeybee, *Apis cerana cerana*, which respectively possess 7, 3 and 11 HSEs [65, 66], are developmentally regulated and stress inducible, but the influence of Hsf1 on the expression of these genes during diapause is unknown. The developmentally regulated sHsps, hsp23 and hsp24 of the Australian sheep blow fly, *Lucilia cuprina*, are induced by heat, and *Lchsp23* possesses a HSE; the role of Hsf in *Lchsp23* expression was not otherwise evaluated [67]. The work described in this study thus constitutes, to the best of our knowledge, the first demonstration that Hsf1 has an important role in regulating gene expression and protein homeostasis during diapause and that for *A*. *franciscana* its influence resides, at least in part, in controlling the synthesis of p26 and artemin. The loss of diapause-specific molecular chaperones was not complete upon reduction of Hsf1 indicating either that sufficient Hsf1 remained to promote gene expression at a reduced level after knockdown, or as shown for αA- and αB-crystallin [49] and Hsps in *Drosophila* [68] the expression of sHsp genes is promoted by transcription factors, such as the forkhead transcription factor FOXO, in addition to Hsf.

The knockdown of Hsf1 by RNAi decreased ArHsp21 and ArHsp22 which are thought to have a limited effect on stress tolerance during diapause of *A*. *franciscana* [26]. The reduction in these proteins suggests that Hsf1 controls genes in *A*. *franciscana* which function other than in stress resistance. In this context, Hsf1 regulates the expression of genes in human cancer cells required for metabolism, the cell cycle and translation [69]. Examination of an *hsf1* mutant of *Saccharomyces cerevisiae* revealed that Hsf1 controls genes needed for the maintenance of cell wall integrity, energy metabolism, protein degradation and carbohydrate metabolism [47]. In other studies Hsf1 regulated genes associated with development, reproduction, metabolism, neurogenesis, carcinogenesis and virulence by way of transcriptional mechanisms that vary from the one employed in stress response [38–43, 49, 50].

At least one other transcription factor regulates diapause and stress tolerance. FOXO, under the influence of juvenile hormone and the insulin signaling pathway, controls the expression of genes encompassing circadian rhythms, fat accumulation, stress resistance, growth control and lifespan during diapause of the mosquito, *Culex pipiens* [70–74]. FOXO, from the cotton bollworm, *Helicoverpa armigera*, binds tightly to the promotor region of the gene that encodes the mRNA *DH-PBAN*, subsequently translated into diapause hormone and the pheromone biosynthesis-activating neuropeptide, both of which regulate development, diapause and pheromone synthesis [75]. Down-regulation of FOXO in *H*. *armigera*, coupled with SUMOylation, decreases diapause hormone and the subsequent initiation of pupal diapause [75]. *FOXO* mRNA is up-regulated in association with diapause in the migratory locust, *Locusta migratoria* L [76] and the ladybird beetle, *Coccinella septempunctata* L [77] but transcriptional regulation by FOXO is yet to be investigated in these organisms.

To summarize, cDNA encoding a structurally typical Hsf1 was cloned from *A*. *franciscana*. The *hsf1* cDNA was used as template to generate dsRNA for the knockdown by RNAi of Hsf1 in nauplii and cysts. Knocking down Hsf1 resulted in premature death of nauplii and lowered the stress tolerance of cysts, the latter in concert with the reduced production of the diapause-specific molecular chaperones p26 and artemin. The findings are consistent with the proposal that Hsf1, in cooperation with other transcription factors, regulates the expression of stress related genes during diapause of *A*. *franciscana*. Moreover, because ArHsp21 and ArHsp22 are reduced in knockdown cysts, Hsf1 influences the expression of diapause-related genes other than those involved overtly in stress tolerance.

## Materials and methods

### Culture of *A*. *franciscana*

*A*. *franciscana* cysts from the Great Salt Lake (INVE Aquaculture Inc., Ogden, UT, USA) were hydrated overnight at 4°C in distilled water, collected by filtration, washed with cold distilled water followed by filtered and autoclaved sea water from Halifax Harbour, hereafter called sea water, and incubated in sea water with vigorous shaking at room temperature. Nauplii were harvested and grown in sea water at room temperature with gentle aeration. Animals were fed daily with *Isochrysis* sp. (clone synonym T-Iso) obtained from the Provasoli-Guillard National Centre for Culture of Marine Phytoplankton (West Boothbay Harbor, ME, USA). The research described in this paper was performed in accordance with the ethical guidelines provided by the Canadian Council on Animal Care (CCAC). The University Committee on Laboratory Animals (UCLA) of Dalhousie University approved the research and assigned the Protocol Number 117–36.

### Cloning of *A*. *franciscana hsf1* cDNA

Five male *A*. *franciscana* adults were heat shocked at 38°C for 20 min and homogenized in an Eppendorf tube with a microfuge pestle (ThermoFisher Scientific, Ottawa, ON, Canada) in TRIzol^®^ (Invitrogen, Burlington, ON, Canada). RNA was recovered with the RiboPure™ RNA Purification Kit (Invitrogen) and cDNA was synthesized with the SuperScript^®^ III First-Strand Synthesis System (Invitrogen) following manufacturer’s instructions.

Primers for the cloning of an *A*. *franciscana hsf1* cDNA were designed by aligning Hsf1 sequences from arthropods deposited with the National Center for Biotechnology Information (NCBI) (Table 2). A partial cDNA encoding *A*. *franciscana* Hsf1 was amplified in PCR mixtures containing 5 μl 10 X Taq buffer, 2 μl 25 mM MgCl_2_, 2 μl dNTP mixture at 10 mM each, 2 μl of each primer at 10 μM, 2 μl first strand cDNA, 34.8 μl H_2_O and 0.2 μl Taq polymerase at 5 U/μl (ThermoFisher Scientific). Amplification was for 5 min at 95°C, followed by 35 cycles of 95°C for 30 s, 49°C for 30 s, 72°C for 1 min and then 6 min at 72°C. PCR products were resolved in 1.2% agarose gels and stained with 0.01% SYBR^®^ Safe (Invitrogen) prior to visualization with a DNR Bio-Imaging Systems MF-ChemiBIS 3.2 gel documentation system (Montreal Biotech, Montreal, QC, Canada). The Frogga Bio 1 kb DNA Ladder (Frogga Bio, North York, ON, Canada) was used as size marker. DNA bands of the appropriate size were excised from the gel and purified with a QIAEX II Gel Extraction Kit (Qiagen, Hilden, Germany). Purified DNA fragments were ligated in the pCR™2.1-TOPO^®^ T/A vector (ThermoFisher Scientific) and used to transform TOP10 competent *Escherichia coli* (ThermoFisher Scientific). cDNA in harvested vectors containing putative *hsf1* cDNA was sequenced at the Center for Applied Genomics DNA Sequencing Facility, Toronto Sick Kids Hospital, Toronto, ON, Canada.

**Table 2:** Primers used for cloning of *hsf1* cDNA by 5’- and 3’-RACE.

Primer Name	Forward Primer (5’- 3’)	Reverse Primer (5’- 3’)	Tm (^o^C)
Partial *hsf1* forward	TAYTTYAARCAY AAYAATATGG	TYYTGRATYTGY TGCTGCTT	49
Partial *hsf1* reverse	TAYTTYAARCAY AAYAATATGG	GCTTCRTTYTC YTTTTTCAT	49
5’-RACE outer	GCTGATGGCGAT GAATGAACACTG*	TCCTCCGATTGT CTTGTACCA	54
5’-RACE inner	CGCGGATCCGAA CACTGCGTTTGC TGGCTTTGATG*	CCTTGCTCAACC GATGATACCTTC CGAAACCC	60
3’-RACE outer	CGGTTGAGCAAG GAAGCCTTC	GCGAGCACAGAA TTAATACGACT*	53
3’-RACE inner	AACGAAAGCTGC CATCTGGTACAA GACAATC	CGCGGATCCGAA TTAATACGACTC ACTCACTATAGG*	60

PCR reaction conditions are described in the Materials and Methods. All primers were synthesized by Integrated DNA Technologies (ODT), Coralville, IA, USA. Tm (^o^C), annealing temperature

*, commercial primers.

Primers for RACE were designed on the basis of the partial sequence of *A*. *franciscana hsf1* cDNA obtained with the degenerative primers (Table 2). cDNA was prepared from *A*. *franciscana* cysts with the FirstChoice^®^ RLM-RACE Kit (Ambion Applied Biosystems, Austin, TX, USA) and used as template to amplify the 5’ and 3’ regions of *hsf1* by nested PCR following manufacturer’s instructions. Outer PCR was at 95°C for 5 min followed by 35 cycles of 95°C for 30 s, 53°C for 30 s, 72°C for 1 min, and then 6 min at 72°C. Inner PCR was at 95°C for 5 min followed by 30 cycles of 95°C for 30 s, 60.5°C for 30 s, 72°C for 1 min and then 6 min at 72°C. PCR products were resolved in agarose gels, stained with 0.01% SYBR^®^ Safe DNA Gel Stain (Invitrogen) and visualized with a DNR Bio-Imaging Systems MF-ChemiBIS 3.2 gel documentation system. PCR products of the appropriate size were excised from the gel and purified with a QIAEX II Gel Extraction Kit (Qiagen, Hilden, Germany), ligated into the pCR™2.1-TOPO^®^ T/A vector (ThermoFisher Scientific) and used to transform TOP10 F’component *E*. *coli* (ThermoFisher Scientific). Putative *hsf1* cDNA fragments were sequenced at the Center for Applied Genomics DNA Sequencing Facility, Toronto Sick Kids Hospital.

### Sequence analysis of Hsf1

The deduced amino acid sequence of *A*. *franciscana* Hsf1 was aligned using Clustal W version 2 (http://www.ebi.ac.uk/Tools/msa/clustalw2/) with the amino acid sequence of Hsf1 from several other organisms available at NCBI. The alignment was presented with DNAMAN (http://www.lynnon.com/dnaman.html).

### Injection of *A*. *franciscana* females with double stranded RNA (dsRNA)

RNA prepared from hydrated *A*. *franciscana* cysts using TRIzol^®^ (Invitrogen) and the RiboPure™ RNA Purification Kit (Invitrogen) was used as described above to make cDNA which was employed for the production of *hsf1* dsRNA with the MEGAscript^®^ RNAi Kit (Ambion Applied Biosystems, Austin, TX, USA) using *hsf1* specific forward 5’-**TAATACGACTCACTATAGG**GAGCCTCGGACTCAC-3’ and reverse 5’-**TAATACGAC TCACTATAGG**GAGTCCAAGACAGG-3’ primers (T7 promoter bold) at 0.2 mM and Platinum *Taq* DNA polymerase (Invitrogen). Touchdown PCR was at 95°C for 5 min, 3 cycles of 95°C for 30 s, 49°C for 30 s and 72°C for 1 min, followed by 3 cycles with the annealing temperature starting at 50°C and successively increased 1°C after each cycle. Subsequently, 30 cycles at 95°C for 30 s, 60°C for 30 s and 72°C for 1 min, followed by 6 min at 72°C were performed. *GFP* dsRNA was amplified from the vector pEGFP-N1 (Clonetech, Mountain View, CA, USA) using the above reaction conditions with Platinum *Taq* DNA polymerase (Invitrogen) and 0.2 mM forward 5’-**TAATACGACTCACTATAGG**GAGACACATGAAGCAGCACGA CCT-3’ and reverse 5’-**TAATACGACTCACTATAGG**GAGAAGTTCACCTTGATGCCCTT C-3’ primers specific to *GFP* (T7 promoters bold) [25, 78]. dsRNAs were resolved in agarose gels and visualized as described above.

*hsf1* and *GFP* dsRNAs were diluted to 0.32 μg/μl with 0.5% phenol red in Dulbecco’s phosphate buffered saline (DPBS) (Sigma-Aldridge, Oakville, ON, Canada). Unfertilized adult *A*. *franciscana* females were injected under an Olympus SZ61 Stereomicroscope (Olympus Canada Inc., Markham, ON, Canada) with 250 nl of solution containing 80 ng of dsRNA for either Hsf1 or GFP. Females were immobilized on 1.6% agarose at 4°C prior to injection with a Nanoject II microinjector (Drummond Scientific Co., Broomall, PA, USA) using glass needles prepared with preset program 33 on a P-97 Flaming/Brown Micropipette Puller (Sutter Instrument Co., Novato, CA, USA) and cut to a sharp angle with a clean razor blade under the stereomicroscope.

### Quantification of *hsf1* and molecular chaperone mRNAs by qRT-PCR

*A*. *franciscana* cysts were harvested within 1 day after release from females injected with dsRNA for either HSF1 or GFP. RNA was extracted from 80 cysts by homogenization with a micropestle (ThermoFisher Scientific) in a 1.5 ml microtube with 100 μl TRIzol^®^ (ThermoFisher Scientific). cDNA was generated with the SuperScript^®^ III First-Strand Synthesis Kit for RT-PCR (ThermoFisher Scientific) using 0.1 μg of RNA as template and oligo dT_20_ primers. All RNA preparations were incubated without reverse transcriptase to ensure the absence of genomic DNA. qPCR was conducted with a QuantiTect^®^ SYBR Green PCR Kit (Qiagen, Mississauga, ON, Canada) in a Rotor-Gene RG-3000 system (Corbett Research, Sydney, NSW, Australia) using 1 μl cDNA and primers specific for HSF1, p26, ArHsp21, ArHsp22, artemin and tyrosinated α-tubulin at 10 μmol/L (Table 3). qPCR was at 50°C for 3 min and then at 95°C for 8 min followed by 30 cycles of 95°C for 20 s, annealing for 20 sec at the temperatures indicated in Table 3 for each mRNA, and 72°C for 40 s. mRNAs for proteins of interest were quantified in duplicate from three independently prepared RNA samples. Copy numbers of *hsf1*, *p26*, *ArHsp21*, *ArHsp22* and *artemin* mRNAs were determined from a standard curve of Ct values and normalized against *α-tubulin* mRNA (King et al., 2013). Primer fidelity was assessed by melting curve analysis.

**Table 3:** Primers used for quantification of mRNAs by qRT-PCR.

Protein Encoded by mRNA	Forward Primer (5’- 3’)	Reverse Primer (5’- 3’)	Tm (^o^C)
Hsf1	GTCCTCCTTGCT TTCGCTATTT	TGTCGGCTTCTT GGTCTGATTC	53
p26	GCACTTAACCCA TGGTACGG	CATCAGATCGCT CGTCATCT	53
ArHsp21	ATCGCTTTTGCT TCTCGG	TTCTGCTCGCAC TTTCCA	52
ArHsp22	GACCCCTTTGCT GACTTA	ATCTGGACGCTC TTTATG	49
Artemin	AGATGCCTTTTC CCATTGTG	CTTGTGAACCGA TGCAGTGT	52
Tubulin	CTGCATGCTGTA CAGAGGAGATGT	CTCCTTCAAGAG AGTCCATGCCAA	53

PCR reaction conditions are described in the Materials and Methods. All primers were synthesized by Integrated DNA Technologies (ODT), Coralville, IA, USA. Tm (^o^C), annealing temperature.

### SDS polyacrylamide gel electrophoresis and immunoprobing of western blots

Fifty cysts released from females injected with either *hsf1* or *GFP* dsRNA were collected by centrifugation for 1 min at 10000 x g, homogenized in 2 x treatment buffer (250 mM Tris, 280 mM SDS, 40% (v/v) glycerol, 5% (v/v) β-mercaptoethanol, 0.2% (w/v) bromophenol blue, pH 6.8), placed in a boiling water bath for 5 min and then centrifuged at 10000 x g for 30 s at room temperature. Protein samples were resolved in 12.5% SDS polyacrylamide gels and either stained with Coomassie blue or blotted at 100 mA overnight at room temperature in transfer buffer (25 mM Tris, 200 mM glycine, 20% (v/v) methanol) to nitrocellulose membranes (Bio-Rad, Mississauga, ON, Canada). The BLUelfpre-stained protein ladder (Frogga Bio) was used as marker.

To detect antibody-reactive proteins, nitrocellulose membranes were incubated at room temperature in TBS (10 mM Tris, 140 mM NaCl, pH 7.4) containing 5% low fat milk for 1 h and then exposed separately in TBS to polyclonal antibodies raised in rabbits against the Hsf1 peptide 525-TLNTPVHAPEAPLFSSRKKK-544 (Abbiotec, San Diego, CA, USA), p26 (Liang and MacRae, 1999), ArHsp21 (Qiu and MacRae, 2008a), ArHsp22 (Qiu and MacRae, 2008b), artemin (Chen et al., 2007) and tyrosinated α-tubulin [79] for 20 min. The membranes were washed sequentially for 1, 2, 3 and 4 min with TBST (TBS containing 0.1% Tween20) followed by 1, 2, 3 and 4 min washes in HST (10 mM Tris, 1 M NaCl, 0.5% Tween20, pH 7.4), with a final wash for 3 min in TBS. Membranes were then incubated for 20 min in HRP-conjugated goat anti-rabbit IgG antibody (Sigma-Aldrich) in TBS. The membranes were washed as described above and immunoreactive proteins were visualized with the Pierce^TM^ ECL western blotting substrate (ThermoFisher Scientific) in a DNR Bio-Imaging Systems MF–ChemiBIS 3.2 gel documentation system.

### Stress tolerance of cysts from *A*. *franciscana* females injected with dsRNA

Cysts released from *A*. *franciscana* females injected with *hsf1* or *GFP* dsRNA were collected separately and incubated in sea water for 7 days to allow entry into diapause. To terminate diapause and simultaneously test stress tolerance these cysts were dried in a desiccator over DryRite (DryRite, Nashville, TN, USA) for 4 weeks and stored at -80°C for 12 weeks (King and MacRae, 2012). The survival of stressed cysts was determined by hatching in seawater at room temperature after which swimming nauplii were counted and removed. Experiments were terminated 5 days after the last nauplius hatched.

That cysts were viable when released from *A*. *franciscana* females injected with dsRNA was determined as described previously using a semi-quantitative assay for metabolism that measured CO_2_ production [25]. Ten cysts from females receiving either *hsf1* or *GFP* dsRNA were incubated immediately after release in 100 μl of test solution (seawater containing 1000 U penicillin, 100 μg/ml streptomycin sulfate and 0.03% phenol red, pH 8.5) in Parafilm sealed Costar 96 well UV plates (Corning Inc., Corning, NY, USA). Control wells contained test solution only. Incubation of cysts in test solution and absorbance measurements at 553 nm (A_553_) were as described [25].

### Viability of nauplii obtained from *A*. *franciscana* females injected with dsRNA

Swimming nauplii released from females injected with either *hsf1* or *GFP* dsRNA were collected within 12 h of release from females and incubated in separate Petri dishes containing seawater at room temperature. The number of surviving nauplii was determined daily for 4 consecutive days with non-motile (dead) nauplii removed as incubation progressed.

### Statistical analysis

A two-tailed student’s t-test (α = 0.05) was used to assess the significant difference between all means from control means for mRNA and protein comparisons. All data were plotted as mean +/- SD unless otherwise stated. Viability of cysts and nauplii after exposing to stressors to terminate diapause was compared using chi-square independence of factors tests. Analyses were carried out using Microsoft Excel 2013.
